# Adherence to a Mediterranean Diet, Body Composition and Energy Expenditure in Outpatients Adolescents Diagnosed with Anorexia Nervosa: A Pilot Study

**DOI:** 10.3390/nu15143223

**Published:** 2023-07-20

**Authors:** Giulia Cinelli, Ileana Croci, Gemma Lou De Santis, Ilenia Chianello, Kiersten Pilar Miller, Paola Gualtieri, Laura Di Renzo, Antonino De Lorenzo, Alberto Eugenio Tozzi, Valeria Zanna

**Affiliations:** 1 Predictive and Preventive Medicine Research Unit, Bambino Gesù Children’s Hospital IRCCS, 00165 Rome, Italy; giulia.cinelli@opbg.net (G.C.); kiersten.millerpilar@opbg.net (K.P.M.); albertoeugenio.tozzi@opbg.net (A.E.T.); 2 PhD School of Applied Medical-Surgical Sciences, University of Rome Tor Vergata, 00133 Rome, Italy; gemmaloudesantis@gmail.com; 3 Anorexia Nervosa and Eating Disorder Unit, Child and Adolescent Neuropsychiatry Unit, Bambino Gesù Children’s Hospital, IRCCS, 00165 Rome, Italy; ilenia.chianello@opbg.net (I.C.); valeria.zanna@opbg.net (V.Z.); 4 Section of Clinical Nutrition and Nutrigenomics, Department of Biomedicine and Prevention, University of Rome Tor Vergata, 00133 Rome, Italy; paola.gualtieri@uniroma2.it (P.G.); laura.di.renzo@uniroma2.it (L.D.R.); delorenzo@uniroma2.it (A.D.L.)

**Keywords:** anorexia nervosa, Mediterranean diet, anthropometrics, body composition

## Abstract

Weight restoration is the primary goal of treatment for patients with Anorexia Nervosa (AN). This observational pilot study aims to describe adherence to the Mediterranean Diet (MD) and the consequent process of weight and functional recovery in outpatient adolescents diagnosed with AN. Eight patients with a median age of 15.1 (14.0–17.1) years were seen at baseline and after six months. Anthropometrics, body composition, and resting energy expenditure (REE) were assessed. The KIDMED questionnaire, the 24 h recall, and a quantitative food frequency questionnaire were used to evaluate adherence to the MD. The median KIDMED score increased from 5.5 (T0) to 10 (T1), which was not significant. Intakes of grams of carbohydrates, lipids, mono-unsaturated fatty acids, and fiber increased (*p* = 0.012, *p* = 0.036, *p* = 0.036, *p* = 0.025). Weight significantly increased (*p* = 0.012) as well as lean body mass (*p* = 0.036), with a resulting improvement of the REE (*p* = 0.012). No association between anthropometrics and body composition and the KIDMED score was found. The MD could represent an optimal dietary pattern for weight gain and nutritional restoration in patients with AN, and it could lead to an improvement in body composition and resting energy expenditure.

## 1. Introduction

Anorexia Nervosa (AN) is an eating disorder with serious physical repercussions and the highest mortality and relapse rate among psychiatric illnesses [1]. The main characteristic of patients with AN is severe food restriction, with an energy intake lower than daily requirements, which leads to significant weight loss and a consequent reduction in both fat and lean body mass [2]. The primary goal of treatment for patients with AN is weight restoration through the refeeding intervention, which is necessary to reduce the risk of physical complications or death [3].

The incidence of AN is increasing among younger girls [1]. In adolescence, many patients do not require hospitalization, and, if necessary, after that, outpatient treatment continues with nutritional rehabilitation managed at home. Based on the clinical experience of the Neuropsychiatry Unit at Bambino Gesù Children’s Hospital, adolescent patients who are not hospitalized may experience differences in the process of refeeding, especially in their food choices. Some patients may reintroduce processed foods, such as snacks or fruit juices, early and with high frequency. While their high-energy-density may help in weight gain, the excess of saturated fatty acids and sugar deviates from the National Guidelines on Healthy Eating recommendations [4].

Body composition is altered in patients with AN. Both fat mass and fat free mass are significantly lower compared to healthy subjects, and they are only partially restored after treatment [5]. Unbalanced nutritional rehabilitation may put patients at risk of metabolic alterations related to dysbiosis of the intestinal microbiome and visceral adiposity. Partial weight gain following a condition of severe physical wasting has, in fact, been associated with a characteristic fat deposition in the abdominal region [5,6,7,8]. This phenomenon may be related to the imbalanced insulin sensitivity detected in AN patients [9], but it has also been linked to potential pathological consequences on the patient’s metabolism, such as insulin resistance [10].

The evaluation of body composition may be of importance to monitor the beneficial effects of a nutritional treatment and to understand both the nature and the course of AN [5]. Improvements in both fat and lean mass have, in fact, been highlighted as markers of menstrual recovery and restoration of health status in the medium and long term [11].

Nutritional rehabilitation should aim to restore health as well as to educate the individual and their family on healthy eating habits, which is essential to ensure the acquisition of the underlying principles needed for a healthy lifestyle. Nutritional education must include restructuring the lifestyle habits of the entire family in order to achieve therapeutic success and prevent complications or relapses in the rehabilitation process. These may include the phenomenon of transitioning from AN to Bulimia Nervosa (BN) and Binge Eating Disorder (BED), which is characterised by events of uncontrolled overeating [12].

The Mediterranean Diet (MD) is characterized by: (i) high intake of complex carbohydrates and fiber from grains, vegetables, and legumes, (ii) moderate consumption of fatty fish and extra-virgin olive oil, and (iii) low consumption of dairy products and meats (red and white) [13]. In the MD, the distribution and quality of macronutrients provide a high amount of monounsaturated fats, fiber, prebiotics, and antioxidants. Population studies on the MD have demonstrated a reduction in the risk of cardiovascular disease, the onset of tumor disease, and insulin resistance in relation to adherence [14,15,16]. An inverse association has been observed between the MD and waist circumference, and, thus, a lower distribution of fat mass at the abdominal level [17,18,19]. Leone et al. suggested that a better adherence to the MD may prevent the occurrence of AN and BN. The hypothesis is that of the overall dietary pattern, tryptophan, unsaturated fatty acids, vitamins, and antioxidant intake may protect against eating disorders [20] and probably against possible relapses.

To date, there are no studies investigating adherence to the MD or its short- or long-term effects in AN patients treated on an outpatient basis. The main aim of the following pilot study is to describe adherence to the MD in a small group of adolescents diagnosed with AN during the outpatient refeeding intervention. Secondly, the study aims to investigate the subsequent process of recovery in terms of weight, body composition, and energy metabolism.

## 2. Materials and Methods

### 2.1. Subjects and Study Design

This observational pilot study analysed data from patients who were admitted to the Neuropsychiatry Unit at Bambino Gesù Children’s Hospital in the period between March 2019 and February 2020 who were diagnosed with anorexia nervosa. Male and female patients, aged 12 to 18 years old, with a primary diagnosis of an eating disorder based on DSM-5 criteria, were invited to take part in the study. Exclusion criteria were the presence of intellectual disabilities and a non-eating disorder primary diagnosis.

All the patients who agreed to participate were enrolled and sent to the Section of Clinical Nutrition and Nutrigenomics, Department of Biomedicine and Prevention of the University of Rome Tor Vergata for nutritional assessment, which included dietary assessment, anthropometrics, body composition, and indirect calorimetry. Visits were performed in the presence of at least one parent or a caregiver. A Mediterranean Diet was prescribed to all the patients, and the complete nutritional follow-up was performed after 3 and 6 months. Adherence to the dietary intervention was monitored every 2 weeks with face-to-face follow-ups or by telephone interviews when needed. All the patients received outpatient psychological treatment throughout the six-month intervention. The study was reviewed and approved by the Ethical Committees of the Bambino Gesù Children Hospital (protocol code 1555_OPBG_2018, 20 November 2018) and was conducted in accordance with the Declaration of Helsinki. All the patients and their parents signed a written informed consent to participate in the study.

### 2.2. Dietary Intervention

All the subjects who participated in the study were given a Mediterranean Diet to follow. The diet was designed to meet the macro- and micronutrient needs of each patient and was personalized according to their preferences. The energy intake was calculated in relation to the BW at T0 as 30–40 kcal/kg/day and increased according to the weight gain in a tailored way for each patient, according to the Italian Ministry Guidelines [21].

The MD was mainly plant based, including seasonal fruits and vegetables, legumes, and nuts. According to the national dietary reference intakes [22], the diet was composed of 45–60% carbohydrates (sugars less than 15%), 20–35% lipids (with less than 10% being saturated fats, 6–10% being polyunsaturated fatty acids, and 15% being monounsaturated fatty acids) and 15–20% proteins. In addition, the daily fiber intake was about 25 g per day.

The diet plan consisted of five meals per day and the distribution of calories across the meals was as follows: breakfast (15–20%), morning snack (5–10%), lunch (35%), afternoon snack (5–10%), and dinner (30%).

### 2.3. Dietary and MD Adherence Assessment

Eating habits were investigated by a trained nutritionist during an interview by using the standard 24 h recall methods and the Italian Version of the medium-length quantitative Food Frequency Questionnaire validated by Buscemi et al. [23]. The “Istituto Scotti Bassani” photo atlas was used to determine the portion sizes and the weight of food consumed [24]. The estimated intake of total daily energy and macronutrients was calculated by using Metadieta dietary software v.4.4 (METEDA S.r.l., Rome, Italy).

The adherence to the MD was assessed using the KIDMED questionnaire for individuals aged 2 to 24 [25]. The questionnaire was administered through an interview conducted by a trained nutritionist. It consisted of 16 questions, with a scoring range of 0 to 12 points. The questions mainly inquired about the frequency of consuming various food groups and their portions, without specifying the quantities in grams. A score of +1 is assigned for positive adherence to the MD, while a score of −1 indicates negative adherence. The sum of the scores was then categorized into three levels: >8 represents “high” adherence to the MD, 4–7 indicates a need for improvement in daily intake according to MD guidelines (“medium”), and ≤3 signifies a lack of conformity with the MD model (“low”).

### 2.4. Anthropometrics

While standing in their underwear, body weight and height were measured using a scale and stadiometer (Invernizzi, Rome, Italy) with precision to the nearest 0.1 kg and 0.1 cm, respectively. Height was measured with the Frankfurt plane of the participant’s head aligned by eye to be parallel to the ground. Body circumferences (waist, abdomen, hip, mid-arm, and mid-thigh) were measured on the non-dominant side using a flexible, non-extensible metric tape. The participant’s body mass index (BMI) was calculated as body weight (in kg) divided by height (in meters) squared. BMI percentile (pBMI) showed how the child’s weight compares to that of other children of the same age and sex, and was determined using the WHO Anthro software v.1.0.4 [26]. In case the software assigned a “Not-Applicable” value to pBMI, a value of 0.1 was used in the analysis by convention.

The International Obesity Task Force (IOTF) childhood BMI cut-offs for thinness, based on and linked to the corresponding adult BMI cut-offs, were used to classify BMI [27,28].

### 2.5. Body Composition

The Bioelectrical Impedance Analysis (BIA) (BIA101S, Akern/RJL Systems, Florence, Italy) was used to measure resistance (Rz) and reactance (Xc). A dedicated piece of software was used to process data and calculate the total body water (TBW, L), the extracellular body water (ECW, L), the body cell mass (BCM) and its index (calculated by dividing it by height (in meters) squared (BCMI)), and the phase angle (PA). 

Dual-energy X-ray absorptiometry (DXA; I-DXA Lunar) was used to assess body composition. Standard quality control and calibration measures were performed before each session, and participants were required to remove any metal jewellery or accessories before the measurement. They were positioned in a supine position with their arms at their sides and their ankles fastened together with a Velcro belt to ensure that they remained in a standard position for approximately 20 min while the DXA scan recorded their results. The coefficient of variation (CV% = 100 × SD/mean) for intra and inter subjects ranged from 1% to 5%, with a coefficient of variation for bone measurements of less than 1%. The radiation dose for the procedure was 0.01 mSv. A dedicated software was used to process data from the body scan and produce measurements for limbs and trunk, including Fat Mass (FM) and Lean Body Mass (LBM). FM% was calculated as FM (in kg) divided by BW.

The Fat Mass Index (FMI) and the Lean Body Mass Index (LBMI) were calculated by dividing the FM and LBM in kilograms by the height in metres squared, respectively. FMI and LBMI were compared to the reference population curve for age and sex [29]. Percentiles between the 10th and the 75th were normal.

### 2.6. Indirect Calorimetry

To determine the resting energy expenditure (REE), indirect calorimetry was conducted using a Vyntus CPX Canopy (CareFusion, Höchberg, Germany) and the Sentry Suite™ software (CareFusion, Höchberg, Germany), according to the method described by De Lorenzo et al. [30]. The gas mixture comprised 12.0% O_2_, 5.0% CO_2_, and N_2_, and the subjects were instructed to fast for 12 h before the measurement. The participants were asked to lie down on a laboratory bed in a supine position for 25–30 min in a room with an ambient temperature of 22 °C until a steady-state condition was achieved, at which point VO_2_ and VCO_2_ values were recorded. The Weir formula [31] was then used to calculate the REE.

### 2.7. Handgrip Strength

The mean handgrip strength of both hands was measured with a portable electronic dynamometer (DynEx, Akern, Florence, Italy). It was regulated for each subject to fit the hand and allow flexion at the metacarpophalangeal joints. The scale of the dynamometer indicated handgrip strength in kilograms (kg). Both non-dominant and dominant hands were measured. The testing protocol consisted of three maximal voluntary isometric contractions maintained for 5 s on both hands, with a rest period of at least 60 s. The mean values for each hand were calculated and considered during the analysis.

During the hand strength testing protocol, the subject sat upright against the back of a chair with feet flat on the floor, and the arm 13 position was standardized with the shoulder adducted and neutrally rotated, with the elbow flexed to 90°. The forearm and wrist were in a neutral position resting on the support surface; the hand was maintained in line with the forearm holding the instrument upright on its base on the short side. Specific verbal instructions were given to subjects before the evaluations, and the experiments were performed with verbal encouragement [32].

### 2.8. Statistical Analysis

Categorical variables were summarized as frequencies and percentages while continuous variables were presented as median and interquartile range (IQR).

The Wilcoxon Signed Rank and the McNemar tests were used to analyze differences between T0 and T1 for continuous and categorical variables, respectively. Spearman’s correlation was applied to test the relationship between anthropometrics and body composition variables and KIDMED score. The Kruscal–Wallis test was used to analyse differences in anthropometrics and body composition variables among the three KIDMED level’s groups.

Data analysis was performed with Stata 17 (Stata Corporation, College Station, TX, USA).

## 3. Results

### 3.1. Subjects

A total of 20 patients were enrolled in the study. Four of them did not give their consent, one was hospitalized before the first visit, and one was not seen for the first visit due to the COVID-19 restrictions. In the end, 14 patients were included in the study, of which only 9 were seen for the follow-up visit at 6 months due to the COVID-19 restrictions. One patient was excluded since she shifted to overeating and was an outlier for weight gain. In the end, eight patients were used for the present analysis. The median age was 15.1 (14.0–17.1) years. At T0, seven patients (87.5%) had secondary amenorrhea since 7.8 (6.2–11.4) months, while 1 (12.5%) was in prepuberty. Two (25%) were in treatment with psychiatric drugs. At T1, menses resumption was observed in four patients over seven (57.1%). The patient in prepuberty did not change her menstrual status at T1.

### 3.2. Dietary Components

The mean dietary components (macronutrients) of the baseline (T0) diet and the follow-up (T1) were calculated using the 24 h recall and the FFQ (Table 1). Only two patients (at T0) and one (at T1) took oral nutritional supplements (200 mL/day corresponding to 300 kcal).

A significant increase in energy intake was observed. A significant increase in carbohydrates and intake of lipids (in grams) was also found, while the intake of proteins increased in grams but decreased in percentage. Sugar intake significantly increased in grams, but the percentage over the total energy intake did not. The intake of both saturated and mono-unsaturated fatty acids significantly increased (in grams). Finally, the amount of fiber significantly increased.

**Figure 1:**
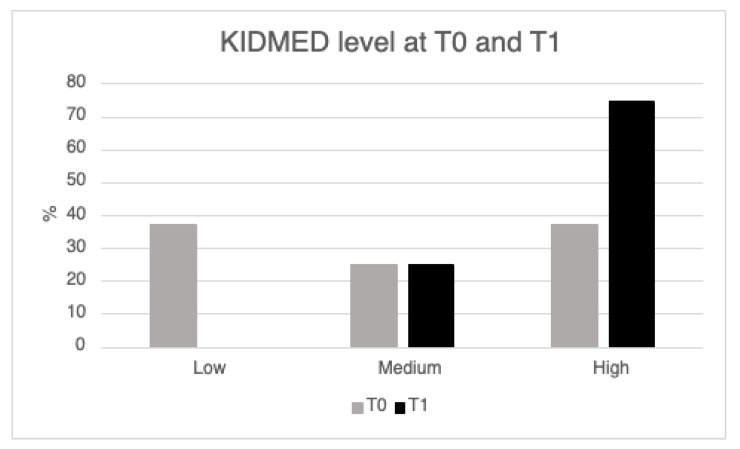
KIDMED level at T0 and T1. Low, medium, and high adherence to the Mediterranean Diet (MD).

**Figure 2:**
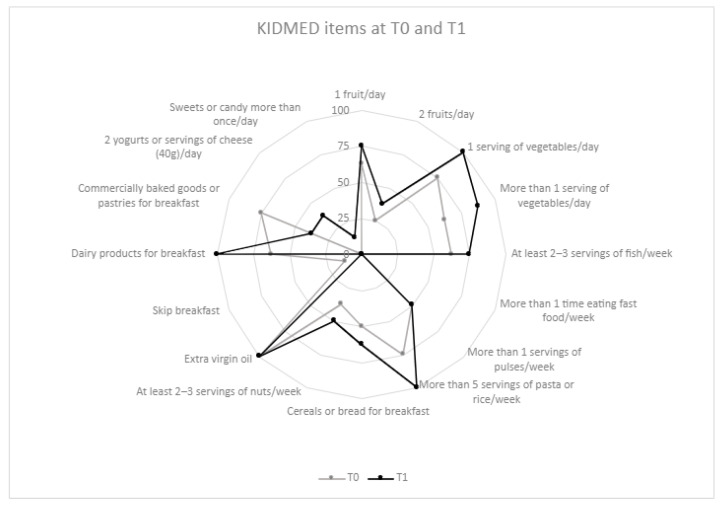
Compliance with items from KIDMED at T0 and T1. The radar chart plots the values of each item of the KIDMED questionnaire along a separate axis that starts in the centre of the chart (0% compliance) and ends at the outer ring (100% compliance). The values are the percentage of the population adherent to each recommendation.

The median KIDMED score at T0 was 5.5 (3.0–8.5) and rose up to 10 (8.0–10.5) at T1 (*p* = 0.457), while the percentage of patients with a “low” MD adherence decreased from 37.5% to none (*p* = 0.083) (Figure 1). An increase in the percentage of patients eating vegetables, fish, grains, and dairy products for breakfast was observed between T0 and T1 (no significant results) (Figure 2). No correlation was found between the KIDMED score at T1 and the energy intake (*p* = 0.663) or the restoration of menses (*p* = 0.882) at T1.

### 3.3. Anthropometry and Body Composition

All anthropometrics improved between T0 and T1 after a mean of 6.7 months between the two visits (Table 2). Complete weight restoration (BMI > 10th percentile) was reached by five patients (62.5%).

No correlation was found between the KIDMED score at T1 and the Δ of weight (r = −0.540, *p* = 0.167) and Δ of BMI percentile (r = −0.417; *p* = 0.304). There was also no correlation between the KIDMED score at T1 and all measured circumferences. When using the KIDMED levels, a significantly higher increase in abdomen and hip circumferences was found in the group of patients with medium adherence compared with the high one (*p* = 0.046; *p* = 0.046).

Regarding body composition, at T1, a significant increase in the total body water, BCM, FFM, LBM, and LBMI at T1 was found. No significant improvement in FM amount was observed, with only three (37.5%) patients showing an FMI over the 10th percentile. A significant improvement in the REE was also detected (Table 3).

No correlation was found between the KIDMED score at T1 and BIA parameters, DXA parameters, and REE. In the DXA analysis, a significant difference between KIDMED medium and high level was found for FM, and, consequently, FMI increased (*p* = 0.045), resulting in a higher gain in the group with poorer MD adherence.

The strength measured through the handgrip increased from 18.7 (17.9–21.0) to 22.5 (20.2–23.9) (*p* = 0.069), and from 18.5 (17.4–20.7) to 21.4 (20.1–22.7) (*p* = 0.093) for the dominant and non-dominant arm, respectively. No correlation between the KIDMED score at T1 and the Δ of the handgrip measurements was found.

## 4. Discussion

The present observational pilot study aimed to describe adherence to a MD in outpatient adolescents diagnosed with AN. At baseline, the MD adherence was “medium”, and it increased to “high” at T1, even if not significant, which demonstrates patients’ compliance to the dietary intervention. The consumption of vegetables, fish, grains, nuts, and dairy products increased. The daily energy intake increased at T1 as well as the amount of all macronutrients and fiber. Conversely, the percentage of calories over the total energy intake from proteins decreased, leading to a better adherence to the national dietary reference values [22]. Finally, sugar intake significantly increased in grams, but the percentage over the total energy intake did not and remained under the recommendation of 15% [22]. Several studies have described how all these dietary characteristics have an antioxidant and anti-inflammatory effect, making the MD an optimal dietary pattern for the prevention and management of nutritional diseases [33,34].

During the six-month intervention, our patients gained weight and improved their BMI percentile as well as all of their anthropometrics, confirming what has been described in previous studies [8,35]. Even though the literature suggests caution in interpreting results of the BIA analysis in AN [36], a significant increase in the TBW was detected in our sample. This could be indicative of an improvement in patients’ hydration status or could be associated with the increased amount of LBM, measured through the DXA analysis.

The circumferences of the upper and lower limbs were significantly increased as an indicator of muscle mass improvement, and this was confirmed by the increased amount of BCM, FFM, and LBM, measured through the BIA and DXA analyses, respectively. These results are in line with a previous study on adolescents [8]. Even though only a trend in the improvement of strength (handgrip) was observed, the improvement of LBM after the nutritional intervention is a clinically important factor [37], and it reflects the consequent increase in REE observed [37]. A recent study by Bou Khalil et al. [38] also showed that the FFM may be a better predictor of REE than weight and BMI. In patients with AN, the FFM is metabolically less active than in other malnourished individuals [38], and it is therefore possible to assume that for the same weight, the REE is lower than in malnourished but non-AN patients. Therefore, the increase in BCM, FFM, and LBM shown by our results may determine a further improvement in the metabolism rehabilitation of these patients.

Additionally, abdomen and hip circumferences, which are regions related to fat deposition, both increased, yet no significant increase in FM measured by the DXA analysis was detected. This contrasts with what was observed by El Ghoch et al. in 33 adolescent patients with AN, in which a significant increase in the total FM was detected. This difference could be related to the fact that their patients were followed for more than three months as inpatients and for only two months as outpatients [8]. The small sample size of the present study could also have affected the significance. When evaluating the waist-to-height ratio, a significant increase was observed. Since this ratio is one of the parameters used to measure central adiposity [39], this observation may confirm the hypothesis that short-term weight recovery in AN adolescent patients could lead to visceral fat redistribution [7,8]. Even if we did not find a significant increase in the total FM, almost 60% of our patients restored their periods.

In our sample, a complete weight recovery was not reached by all patients, with three of them still being underweight after six months. A longer follow-up, which was not possible due to COVID-19 restrictions, could have shown different results in weight and FM gain.

No association between the KIDMED score at T1 and the variation in anthropometrics and body composition parameters was detected. Although when considering the KIDMED level, the group of patients with a poorer adherence to the MD showed a larger increase in FM. Moreover, both abdomen and hip circumferences were higher in patients with poorer adherence. Conversely, there was no association between KIDMED and waist-to-height ratio in our sample. Further investigations with reference methods, such as the assessment of segmental FM in the android and truncal region, on a larger sample are needed to better understand if the MD could represent a dietary pattern reducing the metabolic risk in terms of visceral adiposity. In fact, even if FM restoration is extremely important in AN [40], understanding the amount and the distribution of the regained adipose tissue is essential, since it is related to the metabolic status of the patient [10]. Our main findings were the recovery of weight and major circumferences, confirmed by the improvement in lean mass evidenced by instrumental analysis (BIA and DXA) after the six–month intervention in which MD was chosen for the nutritional rehabilitation. In addition, contrary to what has already been described in the literature, increased dietary adherence resulted in a smaller increase in circumferences related to visceral fat accumulation. These data need to be confirmed with segmental analysis of body masses. Moreover, more than half of our patients have recovered their menstrual cycle. From this perspective, the MD could be a suitable dietary model to propose to patients and family members as it is ideal for good weight recovery, the maintenance of health status, and the prevention of relapses.

The present study has some limitations. Due to COVID-19 restrictions, not all of the enrolled patients were seen at T1 after 6 months, so the final sample size for the analysis was limited. Segmental parameters of body composition were also not available for all patients and, given the small sample size, they were not useful for statistical analysis. However, the novelty of our work is the investigation of MD adherence in AN patients before and after the nutritional rehabilitation.

## 5. Conclusions

This piece of work is an observational pilot study with a small sample size, hence the facts that the results cannot be generalized and it is only possible to speculate on them in order to imagine future research. To our knowledge, this is the first study describing adherence to the MD in a group of outpatient adolescents diagnosed with AN. A MD may be considered an excellent eating model for weight gain and nutritional restoration, even in patients with AN. It could lead to a proper weight restoration as well as an improvement in body composition and resting energy expenditure.

It is also worth highlighting that anthropometrics should not be considered the only aim of treatment of AN. Body composition and FM distribution in relation to the type of dietary pattern are key elements to monitor during the rehabilitation process.

Additional investigations are needed in this field to confirm these results and to better understand FM modification and distribution. The short- and long-term effects of a MD on patients’ metabolism, microbiome, health, and psychological outcomes should be studied.

## Figures and Tables

**Table 1:** Dietary intakes before (T0) and after (T1) the nutritional rehabilitation (n = 8).

	T0	T1	*p*
Energy (Kcal)	1219.1 (587.9–1536.2)	1854.7 (1689.4–2051.6)	**0.025**
Carbohydrates (g)	154.7 (64.5–190.7)	220.2 (210.0–245.4)	**0.012**
Carbohydrates (%)	45.2 (37.4–48.5)	48.1 (44.1–49.1)	0.069
Sugars (g)	47.7 (22.8–62.0)	66.4 (52.8–69.6)	**0.012**
Sugars (%)	13.9 (13.4–16.0)	13.2 (12.0–15.4)	0.161
Proteins (g)	59.6 (35.6–84.8)	87.6 (79.4–95.6)	**0.036**
Proteins (%)	22.8 (21.8–23.8)	19.1 (17.6–20.0)	**0.025**
Animal/plant-based proteins *	2.4 (1.8–3.8)	2.0 (1.6–2.3)	0.124
Lipids (g)	37.6 (24.7–56.4)	70.6 (57.5–83.9)	**0.036**
Lipids (%)	33.5 (30.2–38.4)	32.5 (30.6–36.8)	1.000
Saturated fatty acids (g)	9.6 (6.7–11.8)	21.7 (15.5–25.7)	**0.050**
Saturated fatty acids (%)	8.2 (7.0–12.4)	10.4 (8.4–11.8)	0.327
Mono-unsaturated fatty acids (g)	21.5 (13.4–25.4)	37.5 (27.5–43.0)	**0.036**
Mono-unsaturated fatty acids (%)	17.3 (15.0–19.8)	16.6 (15.9–19.4)	0.575
Poli-unsaturated fatty acids (g)	4.7 (2.3–7.0)	7.3 (6.1–8.0)	0.161
Poli-unsaturated fatty acids (%)	3.5 (3.1–5.2)	3.5 (3.2–3.9)	0.401
Fiber (g)	14.9 (7.2–22.2)	22.6 (20.5–26.5)	**0.025**

* Proteins from the oral nutritional supplements were not included for this variable, since no information about animal and plant-based portions were available. %, percentage of kcal over the total daily energy intake. The Wilcoxon Signed Rank test was performed to compare patients’ dietary intakes at T0 and T1. Statistical significance for *p* < 0.05 (in bold).

**Table 2:** Anthropometric characteristics before (T0) and after (T1) the nutritional rehabilitation (n = 8).

	T0	T1	*p*
Height (cm)	157.3 (153.1–163.9)	158.4 (154.2–165.7)	**0.014**
Weight (kg)	38.1 (36.3–43.0)	46.7 (43.1–49.0)	**0.012**
BMI (kg/m^2^)	15.8 (15.1–16.5)	17.8 (16.9–19.5)	**0.012**
BMI percentile	2.5 (1.2–3.9)	16.8 (5.2–37.8)	**0.012**
Waist circumference (cm)	57.9 (55.2–58.3)	61.5 (59.5–63.2)	**0.025**
Waist-to-height ratio	0.36 (0.35–0.37)	0.38 (0.37–0.40)	**0.036**
Abdomen circumference (cm)	68.9 (63.0–73.1)	71.9 (69.3–75.9)	**0.036**
Hip circumference (cm)	79.3 (76.3–82.0)	87.2 (83.6–89.7)	**0.017**
Mid Harm circumference (cm)	20.4 (19.9–21.0)	23.0 (22.2–24.6)	**0.017**
Mid-thigh circumference (cm)	39.6 (38.6–41.8)	43.0 (42.1–46.7)	**0.017**

Values are expressed as median and IQR (M [IQR]). The Wilcoxon Signed Rank test was performed to compare patients’ anthropometric characteristics at T0 and T1. Statistical significance for *p* < 0.05 (in bold). BMI, body mass index.

**Table 3:** Calorimetry, Bioimpedance Analysis, and Dual X-ray Analysis before and after the nutritional rehabilitation (n = 8).

		T0	T1	*p*
BIA	Rz Resistance	749.5 (711.5–767)	701.5 (682.5–755.5)	0.124
	Xc Reactance	70.0 (66.0–74.5)	69.5 (66.0–78.5)	0.779
	TBW (L)	25.4 (24.1–26.2)	27.0 (25.9–28.1)	**0.012**
	ECW (L)	11.4 (10.7–13.2)	12.0 (11.5–12.9)	0.161
	BCM (kg)	16.3 (15.0–17.2)	17.6 (16.7–21.1)	**0.042**
	BCMI (kg/m^2^)	6.5 (6.1–6.9)	7.4 (6.7–8.0)	0.107
	PA (°)	5.3 (5.1–5.9)	5.8 (5.2–6.3)	0.622
DXA	FFM (kg)	31.59 (29.24–33.07)	34.41 (32.59–37.71)	**0.012**
	FM (kg)	8.57 (6.84–9.63)	10.09 (7.18–12.41)	0.161
	LBM (kg)	31.40 (28.50–34.51)	33.28 (32.00–35.73)	**0.036**
	FMpct (%)	21.2 (17.4–23.8)	21.5 (17.6–26.6)	0.575
	FMI (kg/m^2^)	3.3 (2.9–3.7)	4.0 (2.9–4.9)	0.208
	LBMI (kg/m^2^)	13.0 (11.8–13.0)	13.9 (12.8–14.2)	**0.050**
Calorimetry	VO_2_ (mL/min)	137 (112.5–150.5)	161.5 (148.5–169)	**0.012**
	VCO_2_ (mL/min)	122 (96.5–136)	146.5 (137–157)	**0.035**
	RER	0.88 (0.87–0.96)	0.90 (0.87–0.97)	0.575
	REE (kcal)	921 (781.5–1036.5)	1124.5 (1036–1154.5)	**0.012**

Values are expressed as median and IQR (M [IQR]). The Wilcoxon Signed Rank test was performed to compare patients’ body composition variables at T0 and T1. Statistical significance for *p* < 0.05 (in bold). BIA, bioimpedance analysis; BCM, body cell mass; BCMI, body cell mass index; DXA, dual x-ray analysis; ECW, extracellular water; FFM, fat free mass; FM, fat mass; FMI, fat mass index; FMpct, percentage of fat mass; LBM, lean body mass; LBMI, lean body mass index; PA, phase angle; RER, Respiratory Exchange Ratio; Rz, Resistance; REE, resting energy expenditure; VCO_2_, volumes of carbon dioxide; VO_2_, volumes of oxygen; Xc, Reactance.

## Data Availability

The data presented in this study are available on reasonable request from the corresponding author. The data are not publicly available due to European data sharing policies.
